# Synthesis and Antimicrobial Activity of Some Pyridinium Salts

**DOI:** 10.3390/molecules14125203

**Published:** 2009-12-14

**Authors:** Vildan Alptüzün, Sülünay Parlar, Hüseyin Taşlı, Ercin Erciyas

**Affiliations:** 1 Department of Pharmaceutical Chemistry, Faculty of Pharmacy, Ege University, 35100, Bornova, İzmir, Turkey; E-Mails: sulunay.parlar@ege.edu.tr (S.P.); ercin.erciyas@ege.edu.tr (E.E.); 2 Department of Microbiology, Faculty of Pharmacy, Ege University, 35100 Bornova, İzmir, Turkey; E-Mail: huseyin.tasli@ege.edu.tr (H.T.)

**Keywords:** hydrazones, pyridinium salts, synthesis, antimicrobial activity

## Abstract

Some substituted benzylidenehydrazinylpyridinium derivatives bearing benzyl, ethylphenyl and propylphenyl groups on the pyridinium nitrogen were synthesized and screened for possible antibacterial and antifungal activities against *Staphylococcus aureus*, *Escherichia coli*, *Pseudomonas aeruginosa* and *Candida albicans* using the microdilution method. Antimicrobial test results indicated that compounds containing a 3-phenylpropyl chain displayed the highest antimicrobial activity against *Staphylococcus aureus* and the compound **3d** was the most active in the series against all tested bacteria and fungi strains.

## Introduction

The shortage of new antibacterial drugs and increasing resistance of bacteria to antimicrobial agents are important issues in drug development studies. Quaternized amine derivatives were previously reported to exert antimicrobial properties [1,2,3,4], and several mono- or bis-quaternary ammonium compounds, including various alkyl chain lengths or the same long alkyl chain with different hydrophobic substituents were found to exhibit antimicrobial activity [5,6]. The activities of the quaternary ammonium compounds were attributed to their effect on the cell wall resulting in a direct or indirect lethal effect on the cell viability [7].

Pyridinium halides like quaternary nitrogen salts have antimicrobial properties and adsorption properties on negatively charged solids. The polar heads are cationic pyridiniums, that were related to their antimicrobial activities and characteristics [8,9,10,11,12,13,14,15]. The antimicrobial activity of 1-alkyl-pyridinium salts depends on the adsorptive activities on the surface of bacterial cells as well as their destruction [16] and the pKa values of the corresponding pyridines [17]. Factors controlling their antimicrobial activity are molecular hydrophobicity [18,19], adsorbability [20], surface activity [19] and electron density [21,22] of the ammonium nitrogen atom. These compounds possess one hydrophobic alkyl chain and one hydrophilic quaternary nitrogen ion group in the same molecule, which provides a greater surface activity and more profound antimicrobial potency, compared to conventional antimicrobial agents [23]. 

On the other hand, Schiff bases are important in medicine and many studies have reported their biological activity [24,25]. Hydrazones, a special group of compounds within the Schiff bases, are also known as one of the most important classes of organic compounds, some of which show significant biological activities such as antimicrobial [26,27,28,29,30] antitubercular [31,32,33], anticancer [34,35,36], analgesic [37], anti-inflammatory [37], antiplatelet [38] and antiviral [39] effects. 

**Figure 1:**
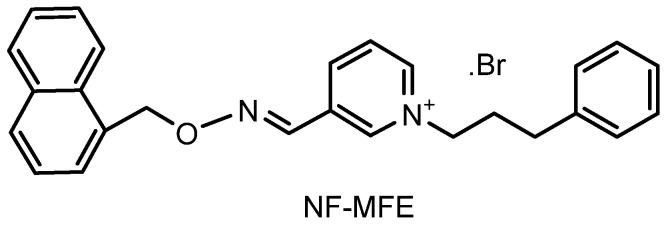
Structure of NF-MFE.

Previously, we synthesized pyridinium oxime-ether derivatives and evaluated them for their antimicrobial activities [40]. The results showed that the R groups attached to the oxime-ether sturucture did not cause a significant change in the antimicrobial effect; however, the side chain attached to the pyridinium nitrogen noticeably affected the antimicrobial activity. Among them, compounds with 3-phenylpropyl chains displayed a remarkable activity and 1-(3-phenylpropyl)-3-[([(naphthyl-1-il)methoxy]imino]methyl]pyridinium bromide (NF-MFE, Figure 1) having a naphthlene ring and a 3-phenylpropyl chain had the lowest MIC values, in other words the highest antimicrobial activity [40].

Based on these findings, *ortho* -methyl, -methoxyl, -hydroxyl benzylidenehydrazinylpyridinium salts with benzyl, 2,6-dichlorobenzyl, 2-phenylethyl and 3-phenylpropyl groups on pyridinium nitrogen were prepared to investigate the effects of such structural modifications of quaternary pyridinium salts on the anticipated antimicrobial activity. It was also planned to replace the oxime-ether function and hydrazone function bioisosterically. Our study also covered the relationship between antimicrobial activity and length of side alkyl chains of the compounds.

## Results and Discussion

### Chemistry

**Scheme 1:**
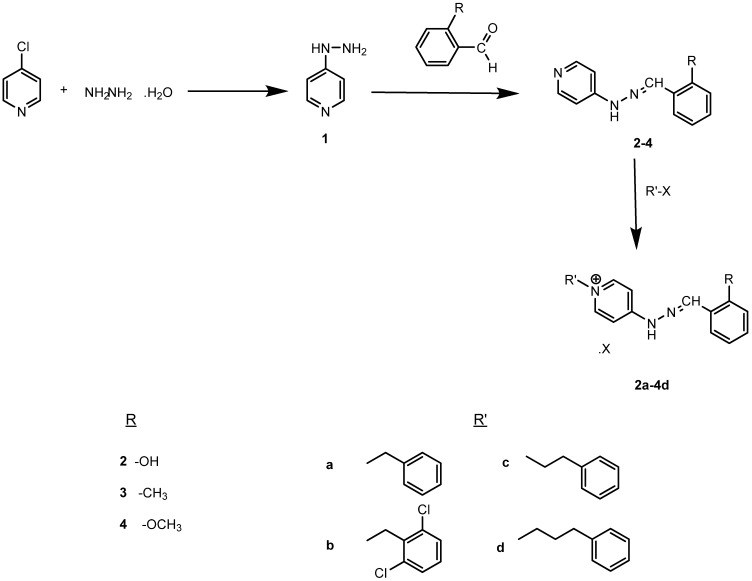
Synthesis of the title compounds.

Benzylidenehydrazinylpyridinium salts were prepared in three steps according to the reported procedure [41], as shown in Scheme 1. In the first step, 4-chloropyridine was refluxed with hydrazine hydrate in 1-propanol to give 4-hydrazinylpyridine (**1**). This compound was then condensed with various substituted aromatic aldehydes in ethanol at room temperature to obtain the corresponding hydrazone derivatives **2-4**. In the last step, the final compounds **2a-4d **were obtained by quaternation of hydrazone derivatives **2-4 **with the appropriate substituted alkyl halide in ethanol under reflux. All title compounds are novel. With the exception of compound **4**, the intermediate compounds **2-4** were reported previously [42,43], but their spectral data have not been described in the literature.

The structures of the final compounds were determined by spectral analyses and the spectroscopic properties were in accord with the proposed structures. The IR spectra of the final compounds showed intense absorption bands within 3,315-3,446 cm^-1^ range and 1,436–1,644 cm^-1^ range that were attributed to NH and C=N function vibrations, respectively. In the ^1^H NMR spectra, the proton signals due to the NH group were recorded between 12.26–13.00 ppm. The proton signals belonging to the N=CH group appeared as singlets at 8.52–8.65 ppm. This chemical shift suggested the *E*-isomer of the compounds. Both IR and ^1^H-NMR data confirmed the condensation between the amino groups and the carbonyl groups. As expected, while the pyridine hydrogens at the 2 and 6 positions of compounds **2**–**4** showed peaks between 8.19–8.21 ppm, the pyridinium hydrogen at these positions in the final compounds **2a**–**4d** showed peaks between 8.30–8.55 ppm [44]. The shifts of these peaks to a higher frequency indicated that compounds **2**–**4** were quaternized with alkyl halide. All the ^13^C-NMR findings confirmed the structures proposed as indicated in the Experimental section. The mass spectra of the final compounds **2a**–**4d **displayed the correct molecular ion peaks.

### Antimicrobial Activity

The new series of benzylidenehydrazinylpyridinium salts with the hydroxyl, methyl, methoxyl substituent in *ortho* position of benzene ring were evaluated for antimicrobial activity toward the Gram positive and Gram negative bacteria and fungus. The bacterial strains represent important Gram positive and Gram negative species, which are *Staphylococcus aureus, Escherichia coli*, and *Pseudomonas aeruginosa*. Their antibacterial activities were assessed by measuring minimum inhibitory concentration (MIC) with standard broth dilution assay (Table 1).

**Table 1:** Antimicrobial activity of compounds **2a**–**4d**.

Compound No	Minimum Inhibitory Concentration (MIC) (μg/mL)			
	*E. coli*	*P. aeruginosa*	*S. aureus*	*C. albicans*
**2a**	64	1024	64	128
**3a**	32	512	16	64
**4a**	64	512	32	128
**2b**	>2,048	>2,048	32	>2,048
**3b**	>2,048	>2,048	64	1,024
**4b**	>2,048	>2,048	128	2,048
**2c**	64	512	32	64
**3c**	32	256	16	64
**4c**	64	512	32	64
**2d**	64	>2,048	8	32
**3d**	32	256	4	32
**4d**	128	1,024	8	64
Ceftazidime	<0.125 (0.06–0.5)*	1 (1–4)*	4 (4–16)*	-
Fluconazole	-	-	-	(0.25–1.0)*

* Acceptable quality control ranges of minimum inhibitory concentrations (MICs) (μg/mL) for reference strains [45,46].

According to the antimicrobial activity results, all compounds were found to possess high antimicrobial activity against *Staphylococcus aureus* and low antimicrobial activity against *Pseudomonas aeruginosa*. Among the tested compounds, **2d**, **3d**, **4d **were found to be the most active derivatives against *Staphylococcus aureus*, being as effective against this organism as the standard compound ceftazidime. The results indicated that the longer the side chain of a compound, the more antimicrobial activity it possesses. Among the benzene ring *ortho*-substitution series, methyl derivatives are more active than the hydroxyl and methoxyl substituted derivatives. In the methyl substituted series, the antimicrobial activity of the phenylpropyl derivative **3d **was followed by the phenylethyl derivative **3c**, benzyl derivative **3a** and dichlorobenzyl derivative **3b**, respectively. No meaningful difference in antibacterial activities were observed for compounds **3c** and **3a**. Both of them had the same MIC values against test microorganisms, except against *Pseudomonas aeruginosa.* According to the MIC values of the compounds, **3d **had lowest MIC values (4 μg/mL) compared to the other pyridinium salts. Moreover, the compounds **2d, 3d, 4d** having the phenylpropyl side chain showed higher activity than oxime-ether derivatives with the same side chain against *Staphylococcus aureus*. This bioisosteric replacement resulted in a slightly increased antimicrobial activity. On the other hand, compounds with a 2,6-dichlorobenzyl side chain on the pyridinium nitrogen displayed no activity against the Gram negative bacteria. Our study revealed that all the compounds had stronger antibacterial activity against Gram positive bacteria when compared to Gram negative bacteria. The findings suggest that the pyridinium salts act on the cell membranes and surface activity of these compounds may be chiefly responsible for the antibacterial properties of the compounds. However, all the tested compounds exhibited low antifungal activity against *Candida albicans*. The reason for the weaker antifungal activity according to antibacterial effect might be postulated as different action in the mechanism of the compounds such as inhibition effect on respiratory systems of fungus cells, rather than cell wall destruction. 

## Experimental

### General

Melting points were determined with an Electrothermal IA9100 melting point apparatus and are not corrected. ^1^H- and ^13^C-NMR spectra were recorded on a Varian AS 400 Mercury Plus NMR instrument. Chemical shifts were measured in DMSO-*d_6_* with TMS as internal reference. Abbreviations for data quoted are: s, singlet; d, doublet; t, triplet; quin, quintet; dd, doublet of doublets; m, multiplet. IR spectra of compounds were recorded as potassium bromide pellets on a Jasco FT/IR-400 spectrometer. The electrospray ionization (ESI) mass spectra were measured on an Agilent 1100 LC/MSD Trap. The conditions of the spray chamber were as follows: ion polarity, positive; drying gas temperature, 300 °C; nebulizer pressure, 10 psi; drying gas flow, 5.00 L min^−1^. Reagents and solvents used for synthesis were purchased from Aldrich, Fluka, and Merck companies. Thin-layer chromatographies were carried out on pre-coated silica gel 60 F_254_ plates (Merck). The spots were visualized with UV light or iodine.

### Synthesis of 4-Hydrazinopyridine Hydrochloride *(**1**)*

Compound **1** was prepared according to the method reported by Mann *et al*. [47]. A solution of 4-chloropyridine (0.01 mol) and hydrazine monohydrate (0.15 mol) in 1-propanol (30 mL) was refluxed for 18 h. The solution was cooled to 0 °C and the precipitate was filtered, washed with cold 1-propanol and crystallized from ethanol. Mp: 242–243 °C (lit. [47,48] 242–243 °C).

### General Procedure for Synthesis of Benzylidenehydrazinylpyridine Derivatives ***2–4***

4-Hydrazinylpyridine (0.01 mol) and the appropriate benzaldehyde derivative (0.01 mol) were stirred in ethanol (30 mL) at room temperature for 5–10 h. The precipitate was filtered and washed with cool ethanol and crystallized from ethanol. 

*4-(2-(2-Hydroxybenzylidene)hydrazinyl)pyridine* (**2**): Yield 58 %; mp: 194 °C; IR (ν, cm^-1^): 1,425, 1,500, 1,542, 1,598 (aromatic C=C and N=C), 3,004 (aromatic C-H), 3,525 (N-H, O-H); ^1^H-NMR δ: 6.84-6.89 (4H, m, Ar-H, pyridine-H), 7.19 (1H, td, *J =* 7.8, 1.6 Hz, Ar-H), 7.64 (1H, dd, *J =* 7.8, 1.6 Hz, Ar-H), 8.20 (2H, d, *J =* 5.9 Hz, pyridine-H), 8.24 (1H, s, N=CH), 10.20 (1H, s, OH), 10.79 (1H, s, NH).

*4-(2-(2-Methylbenzylidene)hydrazinyl)pyridine* (**3**): Yield 65 %; mp: 202 °C IR (cm^-1^): 1,427, 1,452, 1,486, 1,527, 1,538 (aromatic C=C and N=C), 2,886, 2,948 (aliphatic C-H), 3,014 (aromatic C-H), 3,210 (N-H); ^1^H-NMR δ: 6.97 (2H, d, *J =* 6.2 Hz, pyridine-H), 7.19-7.23 (3H, m, Ar-H), 7.83 (1H, d, *J =* 8.2 Hz, Ar-H), 8.19 (1H, s, N=CH), 8.21 (2H, d, *J* = 6.2 Hz, pyridine-H) 10.80 (1H, s, NH).

*4-(2-(2-Methoxybenzylidene)hydrazinyl)pyridine* (**4**): Yield 69 %; mp: 205 °C; IR (ν, cm^-1^): 1,465, 1,492, 1,533, 1,592, (aromatic C=C and N=C), 2,834, 2,886, 2,940, 2,992 (aliphatic C-H), 3,083 (aromatic C-H), 3,212 (N-H); ^1^H-NMR δ: 3.83 (3H, s, -OCH_3_), 6.92 (2H, d, *J =* 6.4 Hz, pyridine-H), 6.98 (1H, t, *J =* 7.2 Hz, Ar-H), 7.05 (1H, d, *J =* 8.0 Hz, Ar-H), 7.32 (1H, td, *J =* 7.8, 1.6 Hz, Ar-H), 7.86 (1H, dd, *J =* 8.0, 1.6 Hz, Ar-H), 8.19 (2H, d, *J =* 6.0 Hz, pyridine-H), 8.29 (1H, s, N=CH), 10.78 (1H, s, NH).

### General Procedure for Synthesis of the Final Compounds ***2a-4d***

A mixture of **2-4 **(0.01 mol) and the corresponding alkyl halide (0.02 mol) were refluxed in ethanol (30 mL) for 6-50 h. The mixture was cooled to room temperature or 0 °C and the obtained precipitate was filtered and washed with cool ethanol. The crude products were crystallized from ethanol to give compounds **2a**–**4d**.

*1-Benzyl-4-(2-(2-hydroxybenzylidene)hydazinyl)pyridinium chloride* (**2a**): Yield 73%; mp: 293 °C; IR (ν, cm^-1^): 1,454, 1,517, 1,552, 1,604 (aromatic C=C and N=C), 1,644 (N^+^=C), 2,844, 2,915 (aliphatic C-H), 3,075 (aromatic C-H), 3,401 (N-H, O-H); ^1^H-NMR δ: 5.46 (2H, s, N^+^-CH_2_-Ph), 6.86 (1H, t, *J =* 7.6 Hz, Ar-H), 6.96 (1H, d, *J =* 8.2 Hz, Ar-H), 7.14 (1H, dd, *J =* 7.0, 2.0 Hz, Ar-H), 7.27 (1H, td, *J =* 7.8, 1.6 Hz, Ar-H), 7.36-7.41 (5H, m, Ar-H, pyridinium-H), 7.55 (1H, dd, *J =* 7.0, 2.3 Hz, pyridinium-H), 7.81 (1H, d, *J =* 7.8 Hz, Ar-H), 8.43 (1H, d, *J =* 7.0 Hz, pyridinium-H), 8.52 (1H, d, *J =* 7.0 Hz, pyridinium-H), 8.64 (1H, s, N=CH), 10.30 (1H, s, OH), 12.93 (1H, s, NH); ^13^C-NMR δ: 60.79 (CH_2_), 107.74 (CH), 109.64 (CH), 117.05 (CH), 120.26 (CH), 120.36 (qC), 126.73 (CH), 129.03 (CH), 129.31 (2 CH), 129.50 (2 CH), 132.77 (CH), 136.20 (qC), 143.62 (CH), 144.84 (CH), 145.82 (CH), 154.17 (qC), 157.57 (qC); ESI-MS m/z: 304.35 (M^+^).

*1-(2,6-Dichlorobenzyl)-4-(2-(2-hydroxybenzylidene)hydrazinyl)pyridinium chloride* (**2b**): Yield 68%; mp: 288 °C; (ν, cm^-1^): 1,440, 1,477, 1,513, 1,548 (aromatic C=C and N=C), 1,643 (N^+^=C), 2,825, 2,892 (aliphatic C-H), 3,062 (aromatic C-H), 3,446 (N-H, O-H); ^1^H-NMR δ: 5.70 (2H, s, N^+^-CH_2_-Ph), 6.87 (1H, t, *J =* 7.8 Hz, Ar-H), 6.94 (1H, d, *J =* 8.4 Hz, Ar-H), 7.06 (1H, dd, *J =* 7.2, 2.4 Hz, pyridinium-H), 7.28 (1H, td, *J =* 7.6, 1.6 Hz, Ar-H), 7.52-7.56 (2H, m, Ar-H, pyridinium-H), 7.64 (2H, d, *J =* 8.0 Hz, Ar-H), 7.80 (1H, dd, *J =* 7.6, 1.6 Hz, Ar-H), 8.17 (1H, d, *J =* 7.6 Hz, pyridinium-H), 8.34 (1H, d, *J =* 7.6 Hz, pyridinium-H), 8.59 (1H, s, N=CH), 10.25 (1H, s, OH), 12.70 (1H, s, NH); ^13^C NMR δ: 56.16 (CH_2_), 107.52 (CH), 109.61 (CH), 117.10 (CH), 120.13 (CH), 120.33 (qC), 126.92 (CH), 130.12 (CH), 130.15 (2 CH), 132.79 (CH), 133.23 (qC), 136.92 (qC), 143.08 (CH), 144.62 (CH), 146.20 (CH), 154.19 (qC), 157.66 (qC); ESI-MS m/z: 372.08 (M^+^), 374.07 (M+2).

*4-(2-(2-Hydroxybenzylidene)hydrazinyl)-1-phenethylpyridinium bromide* (**2c**): Yield 83%; mp: 228 °C; IR (ν, cm^-1^): 1,455, 1,513, 1,550 (aromatic C=C and N=C), 1,644 (N^+^=C), 2,931, 2,971 (aliphatic C-H), 3,070 (aromatic C-H), 3,264 (O-H), 3,415 (N-H); ^1^H-NMR δ: 3.12 (2H, t, *J =* 7.0 Hz, N^+^-CH_2_-CH_2_-Ph), 4.48 (2H, t, *J =* 7.0 Hz, N^+^-CH_2_-CH_2_-Ph), 6.85-6.97 (3H, m, Ar-H, pyridinium-H), 7.19-7.31 (6H, m, Ar-H), 7.49 (1H, dd, *J =* 7.0, 2.3 Hz, pyridinium-H), 7.83 (1H, dd, *J =* 7.8, 1.2 Hz, Ar-H), 8.25 (1H, d, *J =* 7.0 Hz, pyridinium-H), 8.30 (1H, d, *J =* 7.0 Hz, pyridinium-H), 8.56 (1H, s, N=CH), 10.16 (1H, s, OH), 12.27 (1H, s, NH); ^13^C-NMR δ: 36.92 (CH_2_), 59.05 (CH_2_), 107.33 (CH), 109.07 (CH), 117.00 (CH), 120.18 (CH), 120.34 (qC), 126.98 (CH), 127.59 (CH), 129.27 (2 CH), 129.59 (2 CH), 132.79 (CH), 137.34 (qC), 143.58 (CH), 144.28 (CH), 145.53 (CH), 153.96 (qC), 157.48 (qC). ESI-MS m/z: 318.42 (M^+^).

*4-(2-(2-Hydroxybenzylidene)hydrazinyl)-1-(3-phenylpropyl)pyridinium bromide* (**2d**): Yield 41%; mp: 197 °C; IR (ν, cm^-1^): 1,459, 1,492, 1,513, 1,515, 1,552 (aromatic C=C and N=C), 1,644 (N^+^=C), 2,832, 2,904 (aliphatic C-H), 3,031 (aromatic C-H), 3,419 (N-H, O-H); ^1^H-NMR δ: 2.12 (2H, quin, *J =* 7.6 Hz, N^+^-CH_2_-CH_2_-CH_2_-Ph), 2.59 (2H, t, *J =* 8.0 Hz, N^+^-CH_2_-CH_2_-CH_2_-Ph), 4.26 (2H, t, *J =* 7.4 Hz, N^+^-CH_2_-CH_2_-CH_2_-Ph), 6.88 (1H, t, *J =* 7.6 Hz, Ar-H), 6.93 (1H, d, *J =* 8.0 Hz, Ar-H), 7.03 (1H, d, *J =* 6.8 Hz, pyridinium-H), 7.15-7.30 (6H, m, Ar-H), 7.54 (1H, dd, *J =* 7.6, 2.4 Hz, pyridinium-H), 7.84 (1H, dd, *J =* 7.6, 1.6 Hz, Ar-H), 8.37 (1H, d, *J =* 7.2 Hz, pyridinium-H), 8.44 (1H, d, *J =* 7.6 Hz, pyridinium-H), 8.58 (1H, s, N=CH), 10.17 (1H, s, OH), 12.32 (1H, s, NH); ^13^C-NMR δ:32.24 (CH_2_), 32.50 (CH_2_), 58.01 (CH_2_), 107.50 (CH), 109.22 (CH), 117.00 (CH), 120.26 (CH), 120.36(qC), 126.73 (CH), 127.04 (CH), 128.89 (2 CH), 129.09 (2 CH), 132.73 (CH), 141.15 (qC), 143.50 (CH), 144.69 (CH), 145.41 (CH), 153.98 (qC), 157.42 (qC); ESI-MS m/z: 332.18 (M^+^).

*1-Benzyl-4-(2-(2-methylbenzylidene)hydazinyl)pyridinium chloride* (**3a**): Yield 71%; mp: 257 °C; IR (ν, cm^-1^): 1,455, 1,482, 1,517, 1,544, 1,600, (aromatic C=C and N=C), 1,644 (N^+^=C), 2,838, 2,911, 2,979 (aliphatic C-H), 3,037 (aromatic C-H), 3,397 (N-H); ^1^H-NMR δ: 2.45 (3H, s, CH_3_), 5.48 (2H, s, N^+^-CH_2_-Ph), 7.16 (1H, dd, *J =* 7.0, 2.3 Hz, pyridinium-H), 7.28 (1H, t, *J =* 6.6 Hz, Ar-H), 7.33 (1H, d, *J* = 7.8 Hz, Ar-H), 7.36-7.42 (6H, m, Ar-H), 7.56 (1H, d, *J =* 7.4 Hz, pyridinium-H), 7.86 (1H, d, *J =* 7.0 Hz, Ar-H), 8.47 (1H, d, *J =* 7.4 Hz, pyridinium-H), 8.55 (1H, d, *J* = 7.4 Hz, pyridinium-H), 8.62 (1H, s, N=CH), 13.00 (1H, s, NH); ^13^C-NMR δ: 20.13 (CH_3_), 60.75 (CH_2_), 107.80 (CH), 109.64 (CH), 126.96 (CH), 127.47 (CH), 128.78 (2 CH), 129.46 (CH), 129.78 (2 CH), 130.97 (CH), 131.78 (CH), 132.18 (qC), 136.20 (qC), 137.92 (qC), 143.72 (CH), 144.89 (CH), 147.99 (CH), 154.38 (qC); ESI-MS m/z: 302.38 (M^+^). 

*1-(2,6-Dichlorobenzyl)-4-(2-(2-methylbenzylidene)hydrazinyl)pyridinium chloride* **(3b**): Yield 72%; mp: 290 °C; IR (ν, cm^-1^): 1,436, 1,513, 1,581 (aromatic C=C and N=C), 1644 (N^+^=C), 2,694, 2,886 (aliphatic C-H), 3,052 (aromatic C-H), 3,442 (N-H); ^1^H-NMR δ: 2.46 (3H, s, CH_3_), 5.70 (2H, s, N^+^-CH_2_-Ph), 7.14 (1H, dd, *J =* 7.2, 2.8 Hz, pyridinium-H), 7.26-7.30 (2H, m, Ar-H), 7.34 (1H, td, *J =* 7.4, 1.2 Hz, Ar-H), 7.52-7.56 (2H, m, Ar-H, pyridinium-H), 7.64 (2H, d, *J =* 8.0 Hz, Ar-H), 7.84 (1H, dd*, J =* 8.4, 1.6 Hz, Ar-H), 8.22 (1H, dd, *J =* 7.0, 1.6 Hz, pyridinium-H), 8.34 (1H, dd, *J =* 7.2, 1.6 Hz, pyridinium-H), 8.60 (1H, s, N=CH), 13.00 (1H, s, NH); ^13^C-NMR δ: 20.12 (CH_3_) 56.44 (CH_2_), 108.29 (CH), 110.28 (CH), 125.29 (CH), 128.30 (CH), 129.22 (CH), 129.72 (CH), 130.00 (2 CH), 131.74 (CH), 133.16 (qC), 134.16 (qC), 137.00 (qC), 143.50 (CH), 144.66 (CH), 144.74 (CH), 148.69 (qC), 154.80 (qC); ESI-MS m/z: 370.10 (M^+^), 372.09 (M+2).

*4-(2-(2-Methylbenzylidene)hydrazinyl)-1-phenethylpyridinium bromide* **(3c)**: Yield 80%; mp: 190 °C; IR (ν, cm^-1^): 1,455, 1,513, 1,546, 1,577 (aromatic C=C and N=C), 1,644 (N^+^=C), 2,836, 2,908 (aliphatic C-H), 3,054 (aromatic C-H), 3,415 (N-H); ^1^H NMR δ: 2.45 (3H, s, CH_3_), 3.13 (2H, t, *J =* 7.2 Hz, N^+^-CH_2_-CH_2_-Ph), 4.50 (2H, t, *J =* 7.2 Hz, N^+^-CH_2_-CH_2_-Ph), 7.03 (1H, dd, *J =* 7.0, 2.3 Hz, pyridinium-H), 7.19-7.36 (8H, m, Ar-H), 7.50 (1H, dd, *J =* 7.0, 2.3 Hz, pyridinium-H), 7.88 (1H, dd, *J =* 8.2, 1.6 Hz, Ar-H), 8.30 (1H, d, *J =* 7.4 Hz, pyridinium-H), 8.35 (1H, d, *J =* 7.0 Hz, pyridinium-H), 8.53 (1H, s, N=CH), 12.38 (1H, s, NH); ^13^C NMR δ: 20.06 (CH_3_), 36.92 (CH_2_), 59.03 (CH_2_), 107.37 (CH), 108.99 (CH), 126.94 (CH), 127.36 (CH), 127.55 (CH), 129.23 (2 CH), 129.62 (2 CH), 130.96 (CH), 131.73 (CH), 132.12 (qC), 137.33 (qC), 137.89 (qC), 143.71 (CH), 144.84 (CH), 147.53 (CH), 154.10 (qC). ESI-MS m/z: 316.46 (M^+^).

*4-(2-(2-Methylbenzylidene)hydrazinyl)-1-(3-phenylpropyl)pyridinium bromide* (**3d**): Yield 45%; mp: 160 °C; IR (ν, cm^-1^): 1,454, 1,488, 1,511, 1,552, 1,585, (aromatic C=C and N=C), 1,644 (N^+^=C), 2,827, 2,915 (aliphatic C-H), 3,058 (aromatic C-H), 3,424 (N-H); ^1^H-NMR δ: 2.12 (2H, quin, *J =* 7.6 Hz, N^+^-CH_2_-CH_2_-CH_2_-Ph), 2.48 (3H, s, CH_3_), 2.60 (2H, t, *J =* 7.8 Hz, N^+^-CH_2_-CH_2_-CH_2_-Ph), 4.28 (2H, t, *J =* 7.2 Hz, N^+^-CH_2_-CH_2_-CH_2_-Ph), 7.06 (1H, dd, *J =* 7.2, 2.4 Hz, pyridinium-H), 7.16-7.30 (7H, m, Ar-H), 7.35 (1H, td, *J =* 7.2, 1.6 Hz, Ar-H), 7.56 (1H, dd, *J =* 6.8, 2.4 Hz, pyridinium-H), 7.89 (1H, dd, *J =* 7.6, 2.0 Hz, Ar-H), 8.39 (1H, d, *J =* 6.8 Hz, pyridinium-H), 8.46 (1H, d, *J =* 7.2 Hz, pyridinium-H), 8.52 (1H, s, N=CH), 12.34 (1H, s, NH); ^13^C-NMR δ: 20.13 (CH_3_), 32.28 (CH_2_), 52.50 (CH_2_), 58.08 (CH_2_), 107.50 (CH), 109.15 (CH), 126.76 (CH), 126.98 (CH), 127.50 (CH), 128.89 (2 CH), 129.12 (2 CH), 131.00 (CH), 131.80 (CH), 132.14 (qC), 137.33 (qC), 141.15 (qC), 143.79 (CH), 144.80 (CH), 147.64 (CH), 154.29 (qC); ESI-MS m/z: 330.21 (M^+^).

*1-Benzyl-4-(2-(2-methoxybenzylidene)hydazinyl)pyridinium chloride* (**4a**): Yield 73%; mp: 244 °C; IR (ν, cm^-1^): 1,479, 1,513, 1,546, 1,602 (aromatic C=C and N=C), 1,643 (N^+^=C), 2,748, 2,908, 2,971 (aliphatic C-H), 3,037 (aromatic C-H), 3,428 (N-H); ^1^H-NMR δ: 3.85 (3H, s, -OCH_3_), 5.47 (2H, s, N^+^-CH_2_-Ph), 7.02 (1H, t, *J =* 7.4 Hz, Ar-H), 7.11 (1H, d, *J =* 8.2 Hz, Ar-H), 7.16 (1H, dd, *J =* 7.4, 2.7 Hz, pyridinium-H), 7.37-7.46 (6H, m, Ar-H), 7.60 (1H, dd, *J =* 7.2, 2.7 Hz, pyridinium-H), 7.94 (1H, dd, *J =* 7.8, 1.6 Hz, Ar-H), 8.45 (1H, d, *J =* 7.0 Hz, pyridinium-H), 8.54 (1H, d, *J =* 7.0 Hz, pyridinium-H), 8.65 (1H, s, N=CH), 13.00 (1H, s, NH); ^13^C-NMR δ: 56.55 (OCH_3_), 60.82 (CH_2_), 107.96 (CH), 109.76 (CH), 112.84 (CH), 121.52 (CH), 122.04 (qC), 126.49 (CH), 128.72 (2 CH), 129.48 (CH), 129.80 (2 CH), 133.05 (CH), 136.27 (qC), 143.74 (CH), 144.43 (CH), 144.86 (CH), 154.61 (qC), 158.65 (qC); ESI-MS m/z: 318.41 (M^+^).

*1-(2,6-Dichlorobenzyl)-4-(2-(2-methoxybenzylidene)hydrazinyl)pyridinium chloride* (**4b**): Yield 79%; mp: 274 °C; IR (ν, cm^-1^): 1,434, 1,459, 1,479, 1,513, 1,542, 1,602, (aromatic C=C and N=C), 1,641 (N^+^=C), 2,724, 2,904 (aliphatic C-H), 3,064 (aromatic C-H), 3,315 (N-H); ^1^H-NMR δ: 3.86 (3H, s, -OCH_3_), 5.71 (2H, s, N^+^-CH_2_-Ph), 7.01-7.04 (2H, m, Ar-H, pyridinium-H), 7.12 (1H, d, *J =* 8.0 Hz, Ar-H), 7.45 (1H, td, *J =* 7.8, 1.6 Hz, Ar-H), 7.54 (1H, dd, *J =* 8.8, 7.2 Hz, Ar-H), 7.58 (1H, dd, *J =* 7.6, 2.8 Hz, pyridinium-H), 7.64 (2H, d, *J =* 8.0 Hz, Ar-H), 7.91 (1H, dd, *J =* 7.6, 1.6 Hz, Ar-H), 8.17 (1H, d, *J =* 7.2 Hz, pyridinium-H), 8.34 (1H, dd, *J =* 7.2, 1.6 Hz, pyridinium-H), 8.59 (1H, s, N=CH), 12.62 (1H, s, NH); ^13^C-NMR δ: 56.23 (CH_2_), 56.54 (OCH_3_), 107.77 (CH), 109.70 (CH), 112.76 (CH), 121.52 (CH), 122.11 (qC), 130.10 (CH), 130.16 (2 CH), 132.99 (CH), 133.09 (CH), 133.25 (qC), 136.92 (qC), 143.20 (CH), 144.68 (CH), 144.75 (CH), 154.33 (qC), 158.67 (qC). ESI-MS m/z: 386.10 (M^+^), 388.09 (M+2).

*4-(2-(2-Methoxybenzylidene)hydrazinyl)-1-phenethylpyridinium bromide* (**4c**): Yield 95%; mp: 201 °C; IR (ν, cm^-1^): 1,477, 1,513, 1,548, 1,600, (aromatic C=C and N=C), 1,644 (N^+^=C), 2,836, 2,904 (aliphatic C-H), 3,064 (aromatic C-H), 3,421 (N-H); ^1^H-NMR δ: 3.13 (2H, t, *J =* 7.2 Hz, N^+^-CH_2_-CH_2_-Ph), 3.86 (3H, s, -OCH_3_), 4.49 (2H, t, *J =* 7.2 Hz, N^+^-CH_2_-CH_2_-Ph), 6.94 (1H, dd, *J =* 7.2, 2.3 Hz, pyridinium-H), 7.02 (1H, t, *J =* 7.4 Hz, Ar-H), 7.12 (1H, d, *J =* 8.6 Hz, Ar-H), 7.18-7.31 (5H, m, Ar-H), 7.45 (1H, td, *J =* 7.8, 1.2 Hz, Ar-H), 7.54 (1H, dd, *J =* 7.2, 2.3 Hz, pyridinium-H), 7.95 (1H, dd, *J =* 7.6, 1.6 Hz, Ar-H), 8.25 (1H, d, *J =* 7.0 Hz, pyridinium-H), 8.31 (1H, d, *J =* 7.0 Hz, pyridinium-H), 8.57 (1H, s, N=CH), 12.26 (1H, s, NH); ^13^C-NMR δ: 36.92 (CH_2_), 56.57 (OCH_3_), 59.06 (CH_2_), 107.45 (CH), 109.10 (CH), 112.73 (CH), 121.52 (CH), 122.10 (qC), 126.52 (CH), 127.59 (CH), 129.36 (2 CH), 129.61 (2 CH), 132.99 (CH), 137.34 (qC), 143.68 (CH), 144.13 (CH), 144.76 (CH), 154.09 (qC), 158.62 (qC); ESI-MS m/z: 332.46 (M^+^).

*4-(2-(2-Methoxybenzylidene)hydrazinyl)-1-(3-phenylpropyl)pyridinium bromide* (**4d**): Yield 59%; mp: 165 ^°^C; IR (ν, cm^-1^): 1,457, 1,511, 1,546, 1,571, 1,598, (aromatic C=C and N=C), 1644 (N^+^=C), 2,832, 2,908, 2,962 (aliphatic C-H), 3,066 (aromatic C-H), 3,421 (N-H); ^1^H-NMR δ: 2.12 (2H, quin, *J =* 7.6 Hz, N^+^-CH_2_-CH_2_-CH_2_-Ph), 2.59 (2H, t, *J =* 7.8 Hz, N^+^-CH_2_-CH_2_-CH_2_-Ph), 3.86 (3H, s, -OCH_3_), 4.26 (2H, t, *J =* 7.4 Hz, N^+^-CH_2_-CH_2_-CH_2_-Ph), 6.98-7.05 (2H, m, Ar-H, pyridinium-H), 7.12 (1H, d, *J =* 8.0 Hz, Ar-H), 7.16-7.30 (5H, m, Ar-H), 7.45 (1H, td, *J =* 7.6, 1.6 Hz, Ar-H), 7.59 (1H, dd, *J =* 7.0, 2.4 Hz, pyridinium-H), 7.96 (1H, dd, *J =* 7.4, 1.6 Hz, Ar-H), 8.36 (1H, d, *J =* 7.2 Hz, pyridinium-H), 8.44 (1H, d, *J =* 7.6 Hz, pyridinium-H), 8.58 (1H, s, N=CH), 12.30 (1H, s, NH); ^13^C-NMR δ: 32.25 (CH_2_), 32.55 (CH_2_), 56.53 (OCH_3_), 58.08 (CH_2_), 107.64 (CH), 109.25 (CH), 112.65 (CH), 121.51 (CH), 122.11 (qC), 126.46 (CH), 126.72 (CH), 128.89 (2 CH), 129.08 (2 CH), 132.94 (CH), 141.18 (qC), 143.59 (CH), 143.87 (CH), 144.72 (CH), 154.13 (qC), 158.55 (qC); ESI-MS m/z: 346.21 (M^+^).

### Antimicrobial Activity

The minimal inhibitory concentration (MIC) was determined by the broth microdilution method according to the Clinical and Laboratory Standards Institute (CLSI ) [45,46]. *In vitro* antimicrobial activity of the final compounds **2a**–**4d** was evaluated against standard strains; *Staphylococcus aureus* ATCC 29213, *Escherichia coli* ATCC 25922, *Pseudomonas aeruginosa* ATCC 27853 and *Candida albicans* ATCC 90028. The antibacterial and antifungal assay were performed in Mueller–Hinton broth and Sabouraud dextrose broth, respectively. All the synthesized compounds were weighed (10 mg), dissolved in DMSO (250 μL) and diluted with water (750 μL) to prepare the stock solutions of 10 mg/mL. The serial dilution from 2048 to 1 μg/mL was made in a 96-well plate. Fifty μL of a bacterial suspension, obtained from a 24 h culture (~10^6^ cfu/mL) was added to each well with a final DMSO concentration of 1:16. The plates were incubated at 35 °C for 24 h. We tested ceftazidime as antimicrobial agents for quality control of the method. Each experiment was carried out in duplicate.

## Conclusions

In summary, we have described the synthesis of a number of benzylidenehydrazinylpyridinium salts **2a**–**4d **with antimicrobial activity. The final synthesized compounds were characterized by spectral data (IR, ^1^H-NMR, ^13^C-NMR, ESI-MS). Both the synthesis and antimicrobial activity of the final compounds **2a**–**4d** are for the first time. The compounds **2a**–**4d **were found to have reasonable activity against *S. aureus.* A remarkable activity were found in compounds carrying a 3-phenylpropyl side chain on the nitrogen. The most active compound was 4-(2-(2-methylbenzylidene)hydrazinyl)-1-(3-phenylpropyl)pyridinium bromide (**3d**), having MIC value of 4 μg/mL against *S. aureus*. The results indicated that the longer side chain on pyridinium nitrogen caused increased activity. Further investigations of this effect are in progress.
